# Low forearm bone density as a clue to suspect primary hyperparathyroidism in postmenopausal women with osteoporosis: a retrospective study

**DOI:** 10.1007/s11657-026-01665-1

**Published:** 2026-02-08

**Authors:** Ahmet Kürşat Soyer, Ekin Yiğit Köroğlu, Gülsüm Karaahmetli, Didem Özdemir, Oya Topaloğlu, Reyhan Ersoy, Bekir Çakır

**Affiliations:** 1 Department of Endocrinology and Metabolism, Ankara Bilkent City Hospital, Ankara, Turkey; 2 Department of Endocrinology and Metabolism, Ankara Yıldırım Beyazıt University, Ankara, Turkey

**Keywords:** Postmenopausal osteoporosis, Primary hyperparathyroidism, Forearm DXA, One-third distal radius DXA

## Abstract

***Summary*:**

Brief rationale: To evaluate the utility of forearm-DXA in differentiating postmenopausal osteoporosis and to explore its relationship with clinical features of PHPT.

Main result: Forearm-DXA was useful for differential diagnosis and independently associated with calcium in PHPT.

Significance of the paper: This is the first study demonstrating these findings.

**Purpose:**

To evaluate the utility of forearm dual-energy X-ray absorptiometry (DXA) measurement in differentiating primary postmenopausal osteoporosis from primary hyperparathyroidism (PHPT) related osteoporosis and to examine its association with clinical features of PHPT.

**Methods:**

This retrospective study included 246 women with PHPT (153 postmenopausal and 93 premenopausal) and 100 postmenopausal controls with osteopenia/osteoporosis. DXA measurements of the lumbar spine, hip, and one-third distal radius, together with clinical and biochemical data, were compared between postmenopausal patients with PHPT and controls, as well as between postmenopausal and premenopausal PHPT groups. Associations between DXA measurements and clinical findings were also analyzed in PHPT.

**Results:**

Postmenopausal PHPT and controls were matched for age and body-mass index (BMI), osteoporosis was more frequent in PHPT (*p* = 0.001), with the distal radius most affected (*p* < 0.001). Multivariate regression confirmed an independent association between distal radius T-scores and PHPT [OR 1.88, 95% CI: 1.44–2.47]. In postmenopausal patients with osteoporosis by lumbar spine or hip DXA, ROC analysis showed that distal radius scores had excellent discriminatory value (AUC > 0.80) for differentiating PHPT from primary postmenopausal osteoporosis; optimal cutoffs were T-score < −2.4 (80.0% sensitivity, 63.6% specificity) and Z-score < −1.0 (81.5% sensitivity, 63.6% specificity). In premenopausal PHPT, calcium was independently associated with the distal radius T-score [(*B* = −0.594 (95% CI: −1.027 to −0.160)].

**Conclusion:**

This is the first study to show the utility of forearm DXA in differentiating primary postmenopausal osteoporosis from PHPT-related osteoporosis as well as its independent association with calcium in PHPT. Although the diagnosis of PHPT is primarily biochemical, a predominant reduction in distal radius BMD should prompt evaluation for a parathyroid disorder. Moreover, our data may provide clues to help distinguish whether osteoporosis—identified as a surgical indication in PHPT—observed in postmenopausal individuals with PHPT is primarily attributable to PHPT or to menopause.

## Introduction

Parathyroid hormone (PTH) is one of the three key hormones regulating calcium and phosphate homeostasis. Its secretion from the parathyroid glands is modulated by the calcium-sensing receptor (CaSR) in response to serum ionized calcium concentrations. In primary hyperparathyroidism (PHPT), PTH secretion is disproportionately elevated relative to calcium levels [1]. Excessive PTH secretion leads to increased bone resorption, elevated calcitriol synthesis with subsequent rise in intestinal calcium absorption, and greater renal calcium reabsorption [2]. PHPT is most commonly observed between the ages of 50 and 65. Its incidence has been reported as 48.3–50.4 per 100,000 person-years. While the prevalence is similar in men and women under the age of 45, it becomes 3–5 times more frequent in women thereafter [3, 4].

The most common cause of PHPT is the clonal overgrowth of one or more parathyroid glands, accompanied by reduced expression of CaSR within the gland(s) [5]. PHPT is associated with an increased risk of osteoporosis and osteopenia, most prominently affecting cortical bone, such as the distal radius [6], but also involving trabecular sites such as the vertebrae [7]. Consequently, fracture risk is elevated in both cortical- and trabecular-dominant bones [8]. Among patients with PHPT, osteoporosis has been reported in 48.4% and osteopenia in 39.9% [9]. Globally, an estimated nine million osteoporotic fractures occurred in the year 2000, including 1.6 million hip, 1.7 million forearm, and 1.4 million clinical vertebral fractures [10]. Hip or vertebral fractures are known to significantly increase mortality [11]. Postmenopausal women are the most affected group, with reported prevalence rates of 17.4% for osteoporosis and 59.6% for osteopenia [12]. Although PHPT is an important cause of osteoporosis in postmenopausal women, the most common causes of osteoporosis in this population are estrogen deficiency and aging [13]. Postmenopausal primary osteoporosis predominantly affects trabecular bone, which is compromised earlier than cortical bone. This accelerated trabecular resorption is most evident in the vertebral bodies and in the metaphyses of long bones during the early postmenopausal period [14].

Dual-energy X-ray absorptiometry (DXA) is the most widely used technique for assessing bone mineral density (BMD), as it provides highly precise measurements at clinically relevant skeletal sites. A T-score of ≤ −2.5 indicates a substantially increased risk of fracture. DXA measurement is particularly recommended at the spine and hip, as fractures in these regions have a profound impact on patients’ lives [15]. Osteoporosis is usually diagnosed by the presence of a fragility fracture or by DXA measurements at the lumbar spine, total hip, or femoral neck. However, if these regions cannot be reliably evaluated due to conditions such as osteoarthritis or prosthesis, measurement at the one-third distal radius is also accepted for diagnosis [16, 17].

Building on the well-established literature indicating that PHPT predominantly affects cortical bone, whereas postmenopausal primary osteoporosis mainly involves trabecular bone, our study aimed to explore how this distinction can be applied to identify when PHPT should be suspected in postmenopausal women diagnosed with osteoporosis. Moreover, our findings may provide clues to help distinguish whether osteoporosis—identified as a surgical indication in PHPT—observed in postmenopausal individuals with PHPT is primarily attributable to PHPT or to menopause. Finally, our study sought to contribute to the existing literature by examining the relationship between cortical bone loss and other clinical features in patients with PHPT.

## Methods

In this retrospective study, patients were selected from among 2412 women who presented to our endocrinology outpatient clinic between March 2019 and February 2025 and underwent forearm BMD measurement by DXA. Exclusion criteria were as follows: Patients younger than 18 years old, male sex, those without PTH, calcium, phosphorus, or 25-hydroxyvitamin D {25(OH)D] measurements; patients with a history of chronic disease that may affect bone metabolism (rheumatoid arthritis, inflammatory bowel disease, celiac disease, cystic fibrosis, hyperthyroidism, renal disease, or hematologic disorders); patients with a history of smoking or excessive alcohol consumption or who were treated with medications associated with reduced bone mass (e.g., glucocorticoids, cancer chemotherapy, antiseizure drugs); and patients with DXA measurements affected by metallic implants or prostheses. The study group included postmenopausal and premenopausal patients with PHPT, while the control group comprised postmenopausal women with primary osteopenia or osteoporosis. The control group was selected using the same exclusion criteria as the PHPT group, with the exception that all control subjects had normal calcium and PTH levels and no evidence of PHPT. In studies conducted in our country, the mean age at menopause was reported as 47.8 ± 4.0 and 44.38 ± 5.30 years [18, 19]. Accordingly, >55 years was accepted as the postmenopausal age and <40 years as the premenopausal age. Patients aged 40–55 years were excluded from the study.

In patients with classic PHPT, the diagnostic criteria were applied in accordance with the literature: at least two separate measurements of hypercalcemia obtained ≥2 weeks apart, in association with either a frankly elevated PTH or an inappropriately “normal” PTH level. A PTH threshold above 25 pg/mL was accepted [20, 21]. Patients with secondary or tertiary hyperparathyroidism and those with MEN1-related PHPT were also excluded from the PHPT group. Normocalcemic patients with PHPT who were receiving diuretics, lithium, bisphosphonates, or denosumab, as well as those with chronic kidney disease or familial hypocalciuric hypercalcemia (FHH), were excluded to avoid conditions that could mimic a similar presentation. In normocalcemic patients and in those with suspected FHH, 25(OH)D levels were normalized and tests were repeated. All patients had a urinary calcium/creatinine clearance ratio greater than 0.01. In cases with clinical suspicion of FHH, genetic testing was performed when indicated, and FHH was excluded. All patients in the study group met the surgical indications for PHPT according to the Fifth International Workshop guidelines [21] and were selected from among patients whose hyperparathyroidism resolved following surgery.

As a result, 246 patients with PHPT (93 premenopausal and 153 postmenopausal) who met the inclusion criteria were enrolled in the study group, and 100 postmenopausal patients with primary osteopenia or osteoporosis were included in the control group. For all groups, demographic information, laboratory results, and DXA-derived T- and Z-scores of the lumbar spine, total hip, femoral neck, and one-third distal radius were recorded. In the study group, parathyroid and urinary system ultrasonography, as well as 24-h urinary calcium results, were also documented. 99mTechnetium-labeled sestamibi, computerized tomography (CT), or magnetic resonance imaging (MRI) were used when necessary to localize the parathyroid adenoma. The highest serum calcium with concurrent albumin, alkaline phosphatase (ALP), creatinine, phosphorus, 25(OH)D, and intact PTH levels were recorded. In the study group, peak intact PTH was also noted, as well as the highest 24-h urinary calcium value. As ionized calcium measurements were not available for most patients, corrected calcium was calculated both for those with low albumin levels and for patients with values within the normal–high range. The following formula was used: Corrected calcium (mg/dL) = Measured total calcium + [0.8 × (4.0 − albumin (g/dL))]. Normal reference ranges were as follows: calcium, 8.7–10.4 mg/dL; albumin, 3.2–4.8 g/dL; 25(OH)D, >75 nmol/L; phosphorus, 2.4–5.1 mg/dL; creatinine, 0.5–1.1 mg/dL; intact PTH, 18–80 pg/mL; ALP, 53–128 U/L; and eGFR, ≥ 60 mL/min/1.73 m^2^. Threshold value for hypercalciuria was defined as > 250 mg/day. All laboratory tests were carried out on a Roche diagnostic biochemistry analyzer with its original kits.

BMD was assessed at the right femoral neck and total hip, lumbar spine (L1–L4), and one-third distal radius using DXA (Hologic QDR2000, version 5.4; g/cm^2^). DXA calculates BMD (g/cm^2^) by dividing bone mineral content (BMC, g) by bone area (BA, cm^2^). The results were expressed as T-scores and Z-scores. In postmenopausal women, osteoporosis was defined as a history of fragility fracture at the spine, hip, distal radius, humerus, ribs, or pelvis, or as a T-score ≤ –2.5 standard deviation (SD) at either the lumbar spine or hip. In patients for whom spine or hip measurements were not available, the one-third distal radius score was also used for the diagnosis of osteoporosis. Osteopenia was defined as a T-score between −1.0 and −2.5 SD [17]. In premenopausal patients, osteoporosis was defined by a history of low-trauma fracture or the presence of a secondary cause of bone loss, and by either a Z-score ≤ −2.0 or a T-score ≤ −2.5 [22].

All patients with PHPT underwent ultrasonographic evaluation performed by an experienced endocrinologist using a high-frequency linear probe (Hitachi-Hivision-Avius system). The maximum diameter and volume of parathyroid adenomas were calculated from three-dimensional measurements.

Statistical analyses were performed using SPSS software, version 25.0 (SPSS Inc., Chicago, IL, USA). The distribution of the data was evaluated with both graphical approaches and formal tests, including the Kolmogorov–Smirnov and Shapiro–Wilk tests. Variables with normal distribution were compared using the Student’s *t*-test, whereas those without normal distribution were analyzed using the Mann–Whitney *U* test. Continuous variables were expressed as mean ± SD when normally distributed, and as median with interquartile range (IQR) when not normally distributed. Associations between categorical variables were examined with the Chi-square test. Receiver operating characteristic (ROC) curve analyses were carried out to identify optimal cutoff values for T- and Z-scores. The sensitivity and specificity of tests were estimated from the ROC curves. A *p*-value < 0.05 was considered statistically significant. Logistic regression analysis was used to calculate adjusted odds ratios (ORs), accounting for potential confounders, while linear regression was applied for continuous outcomes.

## Results

Among postmenopausal women, there were no significant differences between the PHPT and control groups in terms of age, body mass index (BMI), albumin, or creatinine levels. The PHPT group had higher calcium, corrected calcium, intact PTH, and ALP levels, while 25(OH) vitamin D and phosphorus levels were lower (*p* < 0.001 for each). Osteoporosis was more frequent in the postmenopausal PHPT group (*p* = 0.001). T- and Z-scores at the lumbar spine, hip, and distal radius were all significantly lower in the postmenopausal PHPT group compared with controls. Within the postmenopausal PHPT patients, the distal radius T-scores were significantly lower than both the lumbar spine and femoral neck T-scores (*p* < 0.001) and lumbar spine T-scores were also lower than femoral neck scores (*p* = 0.015). Within the postmenopausal control group, the distal radius T-score was comparable to the lumbar spine but lower than the femoral neck (*p* = 0.267 and 0.048, respectively), with no difference between the lumbar spine and femoral neck (*p* = 0.862) (Table 1).

**Table 1:** Comparison of demographic and clinical characteristics, laboratory and DXA parameters between postmenopausal patients with primary hyperparathyroidism and postmenopausal controls

	**Postmenopausal PHPT (*n*: 154)**	**Postmenopausal control (*n*: 100)**	***p***
Age, years	62 (58–67)	62 (58–68)	0.890
Body mass index, kg/m^2^	27.1 ± 3.3	26.9 ± 2.7	0.548
Calcium, mg/dL	11.56 ± 0.67	9.65 ± 0.42	** <0.001**
Albumin, g/dl	4.53 ± 0.28	4.49 ± 0.32	0.266
Corrected calcium, mg/dL	11.13 ± 0.66	9.25 ± 0.43	** <0.001**
Phosphorus, mg/dL	2.76 ± 0.54	3.68 ± 0.56	** <0.001**
Creatinine, mg/dL	0.74 ± 0.18	0.75 ± 0.12	0.886
Intact PTH, pg/mL	156.5 (116.75–234)	54.7 ± 18.4	** <0.001**
Alkaline Phosphatase	103.5 (79.25–124)	85.2 ± 28.8	** <0.001**
25(OH)D, ng/mL	37 (24–60)	60 (29–78)	** <0.001**
Lumbar spine T-score	−2.05 ± 1.14	−1.55 ± 1.16	**0.001**
Lumbar spine Z score	−0.72 ± 1.09	−0.34 ± 1.23	**0.016**
Femoral neck T-score	−1.83 ± 0.86	−1.55 ± 0.63	**0.004**
Femoral neck Z-score	−0.56 ± 0.88	−0.22 ± 0.81	**0.005**
Total hip T-score	−1.54 ± 0.97	−1.12 ± 0.79	** < 0.001**
Total hip Z-score	−0.72 ± 0.98	−0.33 ± 0.86	**0.002**
Distal radius T-score	−2.83 ± 1.43	−1.76 ± 1.09	** < 0.001**
Distal radius Z-score	−1.58 ± 1.45	−0.51 ± 1.03	** < 0.001**
Osteoporosis (%)	50	28	**0.001**
Fracture history (%)	3.9	8	0.161

Data were expressed as median and 25–75th percentile (P25-P75) and mean ± standard deviation*; DXA,* dual-energy X-ray absorptiometry; *PHPT,* primary hyperparathyroidism*; PTH,* parathyroid hormone; *25(OH)D,* 25-hydroxyvitamin D

In postmenopausal women, univariate analysis showed that lower T-scores at all sites were associated with PHPT. In multivariate analysis, only the distal radius T-score was independently associated with PHPT, regardless of lumbar spine and femoral neck measurements [OR 1.88 (95% CI: 1.44–2.47), *p *< 0.001] (Table 2).

**Table 2:** Univariate and multivariate logistic regression analyses of T-scores in postmenopausal patients for association with primary hyperparathyroidism*

	**Univariate analysis**		Multivariate analysis**	
**Variables**	***p***	**Odds ratio (95% CI)**	***p***	**Odds ratio (95% CI)**
Lumbar spine T-score	**0.005**	**1.41 (1.11–1.78)**	0.67	1.06 (0.80–1.41)
Femoral neck T-score	**0.009**	**1.60 (1.12–2.29)**	0.809	1.05 (0.69–1.60)
Total hip T-score	**0.001**	**1.71 (1.25–2.33)**	–	–
Distal radius T-score	** <0.001**	**1.92 (1.51–2.45)**	** <0.001**	**1.88 (1.44–2.47)**

*CI*, confidence interval

*Analyses were restricted to patients with available DXA measurements at all three skeletal sites (lumbar spine, femoral neck/total hip, and forearm) (*n*: 228)

**Multivariate analysis was performed including lumbar spine, femoral neck, and distal radius T-scores

ROC analysis was performed to assess the value of distal radius DXA in distinguishing PHPT-related osteoporosis from postmenopausal osteoporosis defined by lumbar spine or hip DXA. Accordingly, T- and Z-scores at the distal radius demonstrated excellent discriminatory power (AUC > 0.80). Distal radius T-score < –2.8 yielded 70.3% sensitivity and 81.8% specificity for differentiating PHPT from primary postmenopausal osteoporosis (AUC 0.826, Youden Index 0.521). When a less stringent cutoff of < −2.4 was applied, sensitivity increased to 80.0% with a corresponding specificity of 63.6% (Youden Index 0.564).

Distal radius Z-score < −1.6 yielded 65.6% sensitivity and 90.9% specificity (AUC 0.830, Youden Index 0.565). Using an alternative cutoff of < −1.0 resulted in higher sensitivity (81.5%) at the expense of specificity (63.6%), with a Youden Index of 0.548 (Table 3).

**Table 3:** ROC analysis of forearm scores in differentiating postmenopausal osteoporosis and primary hyperparathyroidism related osteoporosis in postmenopausal osteoporotic patients*

Variables	AUC	95% CI	p	Cutoff	Sensitivity (%)	Specificity (%)	Youden Index
Distal radius T-score	0.826	0.740–0.912	< 0.001	< −2.8	70.3	81.8	0.521
				< −2.4	80.0	63.6	0.564
Distal radius Z-score	0.830	0.744–0.915	< 0.001	< −1.6	65.6	90.9	0.565
				< −1.0	81.5	63.6	0.548

*ROC* receiver operating characteristic, *AUC area under curve, CI confidence interval.*

*From the osteoporotic patients, those with lumbar spine or hip T-scores ≤ −2.5 were selected (*n* = 90)

In the comparison between postmenopausal and premenopausal PHPT patients, the postmenopausal group had higher age, BMI, calcium, corrected calcium, phosphorus, and creatinine levels (Table 4). Albumin, intact PTH, peak intact PTH, 25(OH)D, 24-h urinary calcium excretion, and adenoma size were similar between the groups. In the overall PHPT cohort, the proportion of normocalcemic disease was 13.5%, prevalence of nephrolithiasis was 26.1%, and hypercalciuria was 71.1% and osteoporosis was 40.5%. The proportion of normocalcemic patients, the prevalence of fragility fractures and nephrolithiasis were similar between postmenopausal and premenopausal PHPT patients. Osteoporosis was more frequent in the postmenopausal group. When Z-scores were compared, postmenopausal patients had lower distal radius values, whereas no significant differences were observed in lumbar spine or hip scores (Table 4).

**Table 4:** Comparison of demographic and clinical characteristics, laboratory and DXA parameters between postmenopausal and premenopausal patients with primary hyperparathyroidism

	**Postmenopausal PHPT (*n*: 154)**	**Premenopausal PHPT (*n*: 93)**	***p***
Age, years	62 (58–67)	39 (33–42)	** <0.001**
Body mass index, kg/m^2^	27.1 ± 3.3	24 ± 2.4	** <0.001**
Calcium, mg/dL	11.56 ± 0.67	11.39 ± 0.61	**0.047**
Albumin, gr/dl	4.53 ± 0.28	45.45 ± 2.56	0.641
Corrected calcium, mg/dL	11.13 ± 0.66	10.95 ± 0.61	**0.029**
Normocalcemic (%)	11.1	17.2	0.163
Phosphorus, mg/dL	2.76 ± 0.54	2.56 ± 0.50	**0.005**
Creatinine, mg/dL	0.74 ± 0.18	0.66 ± 0.09	** <0.001**
Intact PTH, pg/mL	156.5 (116.75–234)	168 (121–218)	0.670
Peak Intact PTH, pg/mL	193.0 (137.5–258.5)	204 (154–256)	0.182
Alkaline Phosphatase	103.5 (79.25–124)	94 (76–128)	0.338
25(OH) vitamin D, ng/mL	37 (24–60) b	31 (20–50.5)	0.095
24-h urinary calcium, mg/day	335 (230–471)	340.5 (242–417)	0.884
Hypercalciuria (%)	71	70.8	0.872
Adenoma diameter, mm	12 (9–16)	12.5 (10–17.25)	0.356
Adenoma volume, cm^3^	0.36 (0.16–0.81)	0.31 (0.16–0.76)	0.625
Lumbar spine Z-score	−0.72 ± 1.09	−0.82 ± 0.96	0.477
Femoral neck Z-score	−0.56 ± 0.88	−0.68 ± 0.93	0.263
Total hip Z-score	−0.72 ± 0.98	−0.76 ± 0.96	0.712
Distal radius Z-score	−1.58 ± 1.45	−1.16 ± 1.18	**0.016**
Osteoporosis (%)	50	24.7	** <0.001**
Fracture history (%)	3.9	3.2	1
Nephrolithiasis (%)	26.8	24.7	0.756

*Data were expressed as median and 25–75th percentile (P25-P75) and mean* ± *standard deviation; DXA* dual X-ray energy absorptiometry*, PHPT* primary hyperparathyroidism*, PTH* parathyroid hormone

In postmenopausal patients, distal radius Z-scores were significantly lower than lumbar spine and femoral neck Z-scores (*p* < 0.001 for both), with no significant difference between lumbar spine and femoral neck values (*p* = 0.051). In the premenopausal PHPT group, distal radius Z-score was significantly lower than both the lumbar spine and femoral neck Z-scores (*p* = 0.006 and <0.001, respectively), while lumbar spine and femoral neck Z-scores were comparable (*p* = 0.165).

In premenopausal patients with PHPT, calcium levels correlated moderately with distal radius, and weakly with lumbar spine T-scores. Corrected calcium showed moderate correlation with distal radius. Phosphorus, PTH, peak PTH, and ALP were weakly correlated with distal radius scores. No significant correlations were observed between DXA measurements and 25(OH)D, 24-h urinary calcium, age, or BMI (Table 5).

**Table 5:** Correlation analysis of T-scores with age, BMI, and laboratory findings in premenopausal patients with primary hyperparathyroidism

**Variables**		**Ca**	**C.Ca**	**P**	**PTH**	**Peak PTH**	**ALP**	**25(OH)D**	**24 h Ca**	**Age**	**BMI**
Lumbar spine	*r*	**−0.237**	−0.207	0.137	−0.02	−0.083	−0.183	−0.01	−0.049	0.036	0.085
	*p*	**0.028**	0.056	0.215	0.857	0.45	0.131	0.931	0.654	0.74	0.423
Femoral neck	*r*	−0.087	−0.085	0.072	−0.059	−0.016	−0.037	−0.057	0.141	−0.026	−0.035
	*p*	0.428	0.439	0.517	0.591	0.886	0.765	0.623	0.198	0.811	0.671
Total hip	r	−0.077	−0.05	0.04	0.054	0.107	−0.075	−0.181	0.184	−0.043	0.113
	p	0.484	0.649	0.721	0.624	0.329	0.54	0.113	0.09	0.696	0.335
Distal radius	r	**−0.455**	**−0.409**	**0.275**	**−0.295**	**−0.259**	**−0.322**	0.167	0.007	0.04	0.058
	*p*	** <0.001**	** <0.001**	**0.01**	**0.005**	**0.014**	**0.005**	0.134	0.946	0.707	0.489

*Ca* calcium, *C.Ca* corrected calcium, *P* phosphorus, *PTH* parathyroid hormone, *ALP* alkaline phosphatase, *25(OH)D* 25-hydroxyvitamin D, *24 h Ca* 24-h urinary calcium, *BMI* Body Mass Index

In regression analyses examining the relationship of the distal radius T-score with other variables in premenopausal patients with PHPT, univariate models revealed significant associations with calcium, phosphorus, PTH, and ALP. However, in multivariate analysis, only calcium remained independently associated with the distal radius T-score, irrespective of the other parameters (Table 6).

**Table 6:** Regression analysis of factors associated with distal radius T-scores in premenopausal patients with primary hyperparathyroidism

	**Univariate analysis**		**Multivariate analysis**	
**Variables**	***p***	**B (95% CI)**	***p***	**B (95% CI)**
Calcium, mg/dL	** <0.001**	**−0.882 (−1.248 to −0.516)**	**0.008**	**−0.594 (−1.027 to −0.160)**
Phosphorus, mg/dL	**0.010**	**0.655 (0.163–1.147)**	0.413	0.196 (−0.278–0.670)
Intact PTH, pg/mL	** <0.001**	**−0.004 (−0.005 to −0.002)**	0.172	−0.002 (−0.004–0.001)
Alkaline Phosphatase	** <0.001**	**−0.007 (−0.011 to −0.004)**	0.281	−0.003 (−0.007–0.002)
25(OH)D, ng/mL	0.238	0.008 (−0.005–0.020)	–	–
24-h Ca mg/day	0.826	0.000 (−0.002–0.002)	–	–
Age, years	0.956	−0.001 (−0.038–0.036)	–	–
Body mass index, kg/m^2^	0.470	0.006 (−0.010–0.022)	–	–

*B* unstandardized regression coefficient, *PTH* parathyroid hormone, *25(OH)D*, 25-hydroxyvitamin D, *24 h Ca* 24-h urinary calcium

## Discussion

Although postmenopausal primary osteoporosis is the most common cause of osteoporosis in women of postmenopausal age, PHPT is also responsible for a subset of cases, as it most frequently affects women between 50 and 65 years of age [3, 4]. Cerda et al. reported elevated PTH levels in one-third of postmenopausal women with osteoporosis, and noted that in 10% of these patients the elevation was due to PHPT [23]. Wihlborg et al., in a study of 161 postmenopausal patients with distal forearm fractures, found elevated PTH levels in 20% of the patients and diagnosed PHPT in 6.8% [24]. These two conditions can be distinguished based on clinical history, physical examination, and laboratory tests. However, the detection of PHPT by clinicians is often delayed. Alore et al. [25] reported that only 23.4% of patients with chronic hypercalcemia were evaluated for PHPT. Pajo-Fano et al. [26] found that referral of PHPT patients to endocrinologists took an average of five years, and Asban et al. [27] noted that even among patients with concurrent elevations of calcium and PTH, the diagnosis was missed or delayed in 40% of cases. Additionally, PHPT has a normocalcemic variant, with a reported prevalence of 9–15% in the literature [28, 29]. Although this variant is more difficult to identify through laboratory testing, studies have shown that lumbar spine, hip, and distal radius BMD measurements are similar to those observed in the classic form of the disease [29]. Our findings highlight that the preferential cortical bone loss in PHPT, compared with primary osteoporosis, can serve as a useful discriminator between the two conditions. We also demonstrated its association with other clinical features in PHPT, which has been addressed only in a limited number of previous studies.

In the postmenopausal PHPT group, as expected, calcium, PTH, and ALP levels were higher and phosphorus levels were lower compared with the postmenopausal control group. The prevalence of osteoporosis in the postmenopausal PHPT was 50%, which is lower than the 58.9% reported in the literature for the same group [30]. Consistent with the literature, we observed a higher prevalence of osteoporosis in the postmenopausal PHPT group, with both T- and Z-scores being lower in trabecular- and cortical-rich sites compared with controls. The lumbar spine is predominantly trabecular, the one-third distal radius is predominantly cortical, and the femoral neck contains both trabecular and cortical components. The loss was more pronounced in cortical bone. The literature indicates that in patients with PHPT, cortical-rich sites, particularly the one-third distal radius, are affected earlier and more severely [6, 31]. However, accumulating evidence from advanced skeletal assessment techniques, such as high-resolution peripheral quantitative computed tomography (HR-pQCT) and trabecular bone score (TBS), as well as fracture data, suggests that trabecular bone involvement is also present in PHPT [7, 8, 32]. The findings of our study are consistent with and further support the existing literature. Furthermore, the association between vitamin D deficiency–related secondary hyperparathyroidism and increased cortical porosity [33], as well as the presence of both cortical and trabecular bone loss in chronic kidney disease–related hyperparathyroidism [34], suggests that the bone changes observed in patients with PHPT are largely driven by the effects of elevated PTH. Hyperthyroidism, another cause of osteoporosis, has also been reported to preferentially affect cortical bone, as demonstrated by a study using HR-pQCT [35], whereas glucocorticoid-induced osteoporosis is characterized predominantly by trabecular bone loss [36].

In the postmenopausal control group, the prevalence of osteoporosis was 28%, which is higher than the 17.4% reported in the literature [12]. This may be explained by the exclusion of patients without osteopenia or osteoporosis from our study cohort. The distal radius T-score was comparable to the lumbar spine T-score, but lower than the femoral neck T-score. No significant difference was observed between the lumbar spine and femoral neck T-scores. In the early postmenopausal period, bone loss predominantly occurs in trabecular bone. With advancing age, cortical bone loss also becomes evident, and previous studies have demonstrated that this cortical loss correlates with bone loss in trabecular-rich sites [37]. Furthermore, Miyamura et al. observed that in some patients with postmenopausal primary osteoporosis, the distal radius was more prominently affected, with a correspondingly higher fracture risk at this site [38]. In our study, the finding that the distal radius T-score was comparable to the lumbar spine but lower than the femoral neck T-score supports the observations reported in these two studies [37, 38].

Several studies have addressed the importance of forearm BMD assessment. Biver et al. [39] reported that distal radius measurement provides additional value in evaluating fracture risk. Cauley et al. [40] demonstrated that patients with isolated radial osteoporosis exhibit volumetric and structural deterioration in other skeletal sites, similar to those with hip and lumbar osteoporosis. Cummings et al. [41] found that both distal and proximal radial BMD are strong predictors of hip fractures in elderly women. In postmenopausal women, radial BMD measurement is recommended as a valuable tool for fracture risk assessment, particularly when the hip or spine cannot be reliably evaluated [17]. Although the existing literature underscores the importance of forearm BMD assessment, no studies have investigated how this parameter can be utilized in the differential diagnosis of osteoporosis in postmenopausal women. Identifying PHPT as the underlying cause of osteoporosis in postmenopausal women is of particular importance, as the therapeutic approach differs substantially from that of primary osteoporosis. Timely diagnosis of PHPT is also critical, as osteoporosis prevalence is only 17.1% when identified within one year of hypercalcemia detection, but escalates to 25.4% with delayed recognition [42]. The overall fracture risk is increased 2.01-fold, with forearm and vertebral fracture risks elevated 2.36-fold and 3.00-fold, respectively, in patients with PHPT. Notably, in postmenopausal women with PHPT, vertebral fracture risk may rise up to 8.07-fold [8].

In our regression analysis, we observed that in postmenopausal patients, each 1-SD decrease in the distal radius T-score increased the likelihood of PHPT by 1.88-fold, independently of lumbar spine and femoral neck scores. This finding highlights the independent association between PHPT and the distal radius T-score. In postmenopausal patients with osteoporosis defined by lumbar spine or hip T-scores, our ROC analysis demonstrated the utility of the distal radius T- and Z-scores in differentiating primary osteoporosis from PHPT-related osteoporosis. Distal radius T-score < −2.4 yielded 80.0% sensitivity and 63.6% specificity, while a distal radius Z-score < −1.0 yielded 81.5% sensitivity and 63.6% specificity for this distinction. To the best of our knowledge, this is the first study to report DXA-derived forearm T- and Z-score cutoff values that may aid in the differential diagnosis of these two conditions.

In our comparison of postmenopausal and premenopausal patients with PHPT, we found higher calcium, phosphorus, and creatinine levels in the postmenopausal group. No differences were observed in PTH, ALP, 25(OH) vitamin D levels, adenoma size, or the prevalence of hypercalciuria, nephrolithiasis, and fracture history. Arya et al. [43] reported higher PTH, creatinine, and 25(OH) vitamin D levels in the postmenopausal group compared with the premenopausal group, while lower alkaline phosphatase levels and no significant differences in calcium, phosphorus, 24-h urinary calcium excretion, or adenoma weight. Maldar et al. [44] observed lower ALP and higher creatinine levels in the postmenopausal group, with no significant differences in calcium, phosphorus, 25(OH) vitamin D, or adenoma weight. Castellano et al. [30] found higher phosphorus and creatinine levels in the postmenopausal group, but no differences in PTH, calcium, 25(OH) vitamin D, or urinary calcium excretion. They reported osteoporosis to be more common in the postmenopausal group, whereas nephrolithiasis was more frequent in the premenopausal group. In our study, the higher phosphorus and creatinine levels observed in the postmenopausal group, along with the absence of differences in PTH, 25(OH) vitamin D, and urinary calcium, are consistent with the findings of Castellano et al. [30]. Similarly, the lack of difference in adenoma size aligns with the reports of Arya et al. and Maldar et al. [43, 44]. However, the higher calcium levels in the postmenopausal group represent a novel finding that differs from previous studies. At the end of the discussion, we aim to clarify this observation by presenting forearm DXA measurement.

Consistent with the literature, osteoporosis was more frequently observed in postmenopausal than in premenopausal patients with PHPT [30]. The 24.7% prevalence of osteoporosis observed in premenopausal patients in our study was slightly higher than the 18.5% reported in the literature [30]. Distal radius Z-scores were lower than lumbar spine and femoral neck scores in both postmenopausal and premenopausal patients, supporting the notion that cortical bone is particularly affected in PHPT. The distal radius Z-score was significantly lower in the postmenopausal group, compared to the premenopausal group, whereas no marked differences were observed in lumbar spine or hip Z-scores. Rubin et al. [31] observed that in patients with PHPT, lumbar spine BMD may be preserved for up to 15 years, whereas cortical bone undergoes progressive loss. Castellano et al. [45] found that PHPT patients younger than 65 years had lower lumbar spine and femoral Z-scores compared with those older than 65 years, while no difference was observed between the two groups at the distal radius. They reported that trabecular bone is more affected in patients under 65 years, whereas cortical bone is predominantly affected in those over 65 years. The study by Castellano et al. [45] included male patients as well as both premenopausal and postmenopausal women in the <65-year group, which limits a direct comparison with our findings. In contrast to their results, our study showed similar rather than lower lumbar spine Z-scores in premenopausal patients compared with postmenopausal patients. This may be explained by the absence of menopause-related trabecular loss in our premenopausal group, whereas such an effect was present in the postmenopausal group. Our finding of greater cortical loss in the postmenopausal group corroborates their observation that cortical bone loss becomes more prominent with advancing age.

In our study, to eliminate the confounding effect of menopause, we assessed the relationship between DXA measurements and other parameters only in premenopausal PHPT patients. We found a moderate and statistically significant correlation between serum calcium levels and distal radius scores. Correlations of distal radius scores with phosphorus, PTH, and ALP were weak but statistically significant. In the regression analysis performed to independently assess the relationship between serum calcium levels and the distal radius score, we identified a significant negative association between these two variables, independent of other factors. As previously noted, the higher calcium levels observed in postmenopausal compared with premenopausal patients in our study may be explained by the lower distal radius BMD scores in the postmenopausal group. It is also known that enhanced bone resorption is an important contributor to the development of hypercalcemia in PHPT [2].

Only a limited number of studies have evaluated the relationship between forearm BMD scores and other parameters. Piskinpasa et al. [46] reported a negative correlation between distal radius scores and both corrected calcium and PTH. Walker et al. [47] demonstrated an independent association between distal radius scores and vitamin D. In contrast, Castellano et al. [48] found no significant differences in PTH, calcium, or other biochemical markers between patients with osteoporosis defined solely by distal radius scores and those without. The relationship we observed between forearm scores and both PTH and calcium is consistent with the findings of Piskinpasa et al. [46]. Moreover, to the best of our knowledge, the independent association we identified between calcium levels and the distal radius T-score is novel in the literature. None of the three studies [46–48] evaluated the correlation between forearm scores and ALP or phosphorus levels.

Our study has several limitations. First, being a single-center retrospective study limits the generalizability of the findings. Second, not all patients in the postmenopausal osteoporosis control group had an indication for DXA screening. However, in this group, patients with normal BMD were excluded, and the cutoff values derived from DXA measurements—our most important finding—were studied only for patients with osteoporosis. Third, physical activity status, which may influence bone scores, was not assessed. Fourth, ionized calcium and bone-specific ALP were not measured; however, ALP was not a primary focus of our study. Fifth, more advanced methods for evaluating bone involvement, such as HRpQCT and TBS, were not utilized. Sixth, PHPT patients who did not meet the surgical criteria were not represented in the study group. Seventh, because the ROC analysis was performed in a case–control setting with an enriched prevalence of PHPT, the derived cutoff values should not be interpreted as diagnostic thresholds but rather as adjunctive tools to raise clinical suspicion and guide further biochemical evaluation. Finally, since the menopausal status of patients in our cohort could not be directly verified, women aged 40–55 years were excluded to minimize misclassification. Consequently, the lack of representation of women aged 40–55 years constitutes an important limitation of this study.

In conclusion, to the best of our knowledge, this is the first study to demonstrate the utility of distal radius DXA in the differential diagnosis of osteoporosis in postmenopausal women, providing cutoff values along with their sensitivity and specificity. Furthermore, we also report for the first time an independent association between distal radius T-scores and serum calcium levels in patients with PHPT. Although the diagnosis of PHPT is primarily biochemical, a predominant reduction in distal radius BMD should prompt evaluation for a parathyroid disorder. Moreover, our data may provide clues to help distinguish whether osteoporosis—identified as a surgical indication in PHPT—observed in postmenopausal individuals with PHPT is primarily attributable to PHPT or to menopause.

## Data Availability

The dataset supporting the findings of this study is openly available in Zenodo at 10.5281/zenodo.17114356.

## References

[1] Brown EM (1991) Extracellular Ca2+ sensing, regulation of parathyroid cell function, and role of Ca2+ and other ions as extracellular (first) messengers. Physiol Rev 71(2):371–411. 10.1152/physrev.1991.71.2.371 2006218 10.1152/physrev.1991.71.2.371

[2] Brown EM (2013) Role of the calcium-sensing receptor in extracellular calcium homeostasis. Best Pract Res Clin Endocrinol Metab 27(3):333–343. 10.1016/j.beem.2013.02.006 23856263 10.1016/j.beem.2013.02.006

[3] Yeh MW, Ituarte PHG, Zhou HC et al (2013) Incidence and prevalence of primary hyperparathyroidism in a racially mixed population. J Clin Endocrinol Metab 98(3):1122–9. 10.1210/jc.2012-4022 23418315 10.1210/jc.2012-4022 PMC3590475

[4] Minisola S, Arnold A, Belaya Z et al (2020) Epidemiology, pathophysiology, and genetics of primary hyperparathyroidism. J Bone Miner Res 37(11):2315–29. 10.1002/jbmr.4665 10.1002/jbmr.4665 PMC10092691 36245271

[5] Singh P, Bhadada SK, Dahiya D et al (2020) Reduced calcium sensing receptor (CaSR) expression is epigenetically deregulated in parathyroid adenomas. J Clin Endocrinol Metab 105(9):3015–24. 10.1210/clinem/dgaa419 32609827 10.1210/clinem/dgaa419 PMC7500582

[6] Silverberg SJ, Shane E, De La Cruz L et al (1989) Skeletal disease in primary hyperparathyroidism. J Bone Miner Res 4(3):283–91. 10.1002/jbmr.5650040302 2763869 10.1002/jbmr.5650040302

[7] Stein EM, Silva BC, Boutroy S et al (2013) Primary hyperparathyroidism is associated with abnormal cortical and trabecular microstructure and reduced bone stiffness in postmenopausal women. J Bone Miner Res 28(5):1029–40. 10.1002/jbmr.1841 23225022 10.1002/jbmr.1841 PMC3631282

[8] Ejlsmark-Svensson H, Rolighed L, Harsløf T, Rejnmark L (2021) Risk of fractures in primary hyperparathyroidism: a systematic review and meta-analysis. Osteoporos Int 32(6):1053–1060. 10.1007/s00198-021-05822-9 33527175 10.1007/s00198-021-05822-9

[9] Reid LJ, Muthukrishnan B, Patel D, Seckl JR, Gibb FW (2019) Predictors of nephrolithiasis, osteoporosis, and mortality in primary hyperparathyroidism. J Clin Endocrinol Metab 104(9):3692–3700. 10.1210/jc.2018-02483 30916764 10.1210/jc.2018-02483

[10] Johnell O, Kanis JA (2006) An estimate of the worldwide prevalence and disability associated with osteoporotic fractures. Osteoporos Int 17(12):1726–1733. 10.1007/s00198-006-0172-4 16983459 10.1007/s00198-006-0172-4

[11] Ioannidis G, Papaioannou A, Hopman WM et al (2009) Relation between fractures and mortality: results from the Canadian Multicentre Osteoporosis Study. CMAJ 181(5):265–71. 10.1503/cmaj.081720 19654194 10.1503/cmaj.081720 PMC2734204

[12] Zhang X, Wang Z, Zhang D et al (2023) The prevalence and treatment rate trends of osteoporosis in postmenopausal women. PLoS One 18(9):e0290289. 10.1371/journal.pone.0290289 37751427 10.1371/journal.pone.0290289 PMC10522039

[13] Ebeling PR, Nguyen HH, Aleksova J, Vincent AJ, Wong P, Milat F (2022) Secondary osteoporosis. Endocr Rev 43(2):240–313. 10.1210/endrev/bnab028 34476488 10.1210/endrev/bnab028

[14] Seeman E (2008) Bone quality: the material and structural basis of bone strength. J Bone Miner Metab 26(1):1–8. 10.1007/s00774-007-0793-5 18095057 10.1007/s00774-007-0793-5

[15] Cummings SR, Bates D, Black DM (2002) Clinical use of bone densitometry. JAMA 288(15):1889. 10.1001/jama.288.15.1889 12377088 10.1001/jama.288.15.1889

[16] Binkley N, Bilezikian JP, Kendler DL, Leib ES, Lewiecki EM, Petak SM (2006) Official positions of the International Society for Clinical Densitometry and Executive Summary of the 2005 Position Development Conference. J Clin Densitom 9(1):4–14. 10.1016/j.jocd.2006.05.002 16731426 10.1016/j.jocd.2006.05.002

[17] Siris ES, Adler R, Bilezikian J et al (2014) The clinical diagnosis of osteoporosis: a position statement from the National Bone Health Alliance Working Group. Osteoporos Int 25(5):1439–43. 10.1007/s00198-014-2655-z 24577348 10.1007/s00198-014-2655-z PMC3988515

[18] Neslihan Carda S, Bilge SA, Öztürk TN, Oya G, Ece O, Hamiyet B (1998) The menopausal age, related factors and climacteric symptoms in Turkish women. Maturitas 30(1):37–40. 10.1016/s0378-5122(98)00041-3 9819781 10.1016/s0378-5122(98)00041-3

[19] Biri A, Bakar C, Maral I, Karabacak O, Bumin MA (2005) Women with and without menopause over age of 40 in Turkey: consequences and treatment options. Maturitas 50(3):167–176. 10.1016/j.maturitas.2004.05.013 15734597 10.1016/j.maturitas.2004.05.013

[20] Silva BC, Cusano NE, Bilezikian JP (2024) Primary hyperparathyroidism. Best Pract Res Clin Endocrinol Metab 38(1):101247. 10.1016/j.beem.2018.09.013 30477754 10.1016/j.beem.2018.09.013

[21] Bilezikian JP, Khan AA, Silverberg SJ et al (2020) Evaluation and management of primary hyperparathyroidism: summary statement and guidelines from the Fifth International Workshop. J Bone Miner Res 37(11):2293–314. 10.1002/jbmr.4677 10.1002/jbmr.4677 36245251

[22] Pepe J, Body J-J, Hadji P et al (2020) Osteoporosis in premenopausal women: a clinical narrative review by the ECTS and the IOF. J Clin Endocrinol Metab 105(8):2487–506. 10.1210/clinem/dgaa306 10.1210/clinem/dgaa306 32453819

[23] Cerdà D, Peris P, Monegal A et al (2011) Aumento de los valores de PTH en la mujer con osteoporosis posmenopáusica. Rev Clin Esp 211(7):338–43. 10.1016/j.rce.2011.03.014 21596374 10.1016/j.rce.2011.03.014

[24] Wihlborg A, Bergström K, Gerdhem P, Bergström I (2022) Parathyroid hormone disturbances in postmenopausal women with distal forearm fracture. World J Surg 46(1):128–135. 10.1007/s00268-021-06331-w 34647149 10.1007/s00268-021-06331-w PMC8677684

[25] Alore EA, Suliburk JW, Ramsey DJ et al (2019) Diagnosis and management of primary hyperparathyroidism across the Veterans Affairs Health Care System. JAMA Intern Med 179(9):1220. 10.1001/jamainternmed.2019.1747 31305864 10.1001/jamainternmed.2019.1747 PMC6632180

[26] Paja-Fano M, Martínez-Martínez A-L, Monzón-Mendiolea A (2020) Retraso diagnóstico y terapéutico en el hiperparatiroidismo primario. Un problema no resuelto. Endocrinología, Diabetes y Nutrición 67(6):357–363. 10.1016/j.endinu.2019.11.002 31982385 10.1016/j.endinu.2019.11.002

[27] Asban A, Dombrowsky A, Mallick R et al (2019) Failure to diagnose and treat hyperparathyroidism among patients with hypercalcemia: opportunities for intervention at the patient and physician level to increase surgical referral. Oncologist 24(9):e828–e34. 10.1634/theoncologist.2018-0424 31019019 10.1634/theoncologist.2018-0424 PMC6738291

[28] Pandian TK, Lubitz CC, Bird SH, Kuo LE, Stephen AE (2020) Normocalcemic hyperparathyroidism: a collaborative Endocrine Surgery Quality Improvement Program analysis. Surgery 167(1):168–172. 10.1016/j.surg.2019.06.043 31543325 10.1016/j.surg.2019.06.043

[29] Yankova I, Lilova L, Petrova D et al (2024) Biochemical characteristics and clinical manifestation of normocalcemic primary hyperparathyroidism. Endocrine 85(1):341–6. 10.1007/s12020-024-03768-6 38489132 10.1007/s12020-024-03768-6

[30] Castellano E, Attanasio R, Boriano A et al (2017) Sex difference in the clinical presentation of primary hyperparathyroidism: influence of menopausal status. J Clin Endocrinol Metab 102(11):4148–52. 10.1210/jc.2017-01080 28938410 10.1210/jc.2017-01080

[31] Rubin MR, Bilezikian JP, McMahon DJ et al (2008) The natural history of primary hyperparathyroidism with or without parathyroid surgery after 15 years. J Clin Endocrinol Metab 93(9):3462–70. 10.1210/jc.2007-1215 18544625 10.1210/jc.2007-1215 PMC2567863

[32] Arboiro-Pinel R, Mahíllo-Fernández I, Díaz-Curiel M (2022) Bone analysis using trabecular bone score and dual-energy X-ray absorptiometry-based 3-dimensional modeling in postmenopausal women with primary hyperparathyroidism. Endocr Pract 28(1):83–89. 10.1016/j.eprac.2021.08.006 34450273 10.1016/j.eprac.2021.08.006

[33] Sundh D, Mellström D, Ljunggren Ö et al (2016) Low serum vitamin D is associated with higher cortical porosity in elderly men. J Intern Med 280(5):496–508. 10.1111/joim.12514 27196563 10.1111/joim.12514

[34] Ruderman I, Rajapakse CS, Opperman A et al (2020) Bone microarchitecture in patients undergoing parathyroidectomy for management of secondary hyperparathyroidism. Bone Rep 13:100297. 10.1016/j.bonr.2020.100297 32760761 10.1016/j.bonr.2020.100297 PMC7393533

[35] Nicolaisen P, Obling ML, Winther KH et al (2021) Consequences of hyperthyroidism and its treatment for bone microarchitecture assessed by high-resolution peripheral quantitative computed tomography. Thyroid 31(2):208–16. 10.1089/thy.2020.0084 32703114 10.1089/thy.2020.0084

[36] Briot K, Roux C (2015) Glucocorticoid-induced osteoporosis. RMD Open 1(1):e000014. 10.1136/rmdopen-2014-000014 26509049 10.1136/rmdopen-2014-000014 PMC4613168

[37] Ruotsalainen T, Panfilov E, Thevenot J et al (2025) Total radius BMD correlates with the hip and lumbar spine BMD among post-menopausal patients with fragility wrist fracture in a machine learning model. Arch Osteoporos. 10.1007/s11657-025-01542-3 40366492 10.1007/s11657-025-01542-3

[38] Miyamura S, Kuriyama K, Ebina K et al (2020) Utility of distal forearm DXA as a screening tool for primary osteoporotic fragility fractures of the distal radius. JBJS Open Access 5(1):e0036. 10.2106/jbjs.oa.19.00036 32309758 10.2106/JBJS.OA.19.00036 PMC7147634

[39] Biver E, Durosier-Izart C, Chevalley T, Van Rietbergen B, Rizzoli R, Ferrari S (2018) Evaluation of radius microstructure and areal bone mineral density improves fracture prediction in postmenopausal women. J Bone Miner Res 33(2):328–337. 10.1002/jbmr.3299 28960489 10.1002/jbmr.3299

[40] Cauley JA, Lui L-Y, Leboff MS, Watts NB (2024) New challenges: use and interpretation of radius bone mineral density. J Clin Endocrinol Metab 110(1):e1–e7. 10.1210/clinem/dgae726 39403961 10.1210/clinem/dgae726 PMC11651675

[41] Cummings SR (1990) Appendicular bone density and age predict hip fracture in women. JAMA 263(5):665. 10.1001/jama.1990.03440050059033 2404146

[42] Lorenz FJ, Beauchamp-Perez F, Manni A, Chung T, Goldenberg D, Goyal N (2022) Analysis of time to diagnosis and outcomes among adults with primary hyperparathyroidism. JAMA Netw Open 5(12):e2248332. 10.1001/jamanetworkopen.2022.48332 36574247 10.1001/jamanetworkopen.2022.48332 PMC9857508

[43] Arya AK, Bhadada SK, Kumari P et al (2020) Differences in primary hyperparathyroidism between pre- and postmenopausal women in India. Endocr Pract. 10.1016/j.eprac.2020.12.012 33685668 10.1016/j.eprac.2020.12.012

[44] Maldar AN, Shah NF, Chauhan PH, Lala M, Kirtane MV, Chadha M (2023) Differences in the presentation and outcome between premenopausal and postmenopausal primary hyperparathyroidism Indian women: a single-center experience. J Midlife Health 14(2):73–80. 10.4103/jmh.jmh_142_22 38029031 10.4103/jmh.jmh_142_22 PMC10664047

[45] Castellano E, Attanasio R, Boriano A, Borretta G (2019) Clinical presentation of primary hyperparathyroidism in older adults. J Endocr Soc 3(12):2305–2312. 10.1210/js.2019-00316 31745527 10.1210/js.2019-00316 PMC6853663

[46] Piskinpasa H, Dogansen SC, Metin D et al (2023) The significance of forearm bone mineral density evaluation in determining surgical indications in primary hyperparathyroidism. Ann Endocrinol (Paris) 84(1):8–13. 10.1016/j.ando.2022.08.004 36252847 10.1016/j.ando.2022.08.004

[47] Walker MD, Cong E, Lee JA et al (2015) Vitamin D in primary hyperparathyroidism: effects on clinical, biochemical, and densitometric presentation. J Clin Endocrinol Metab 100(9):3443–51. 10.1210/jc.2015-2022 26079779 10.1210/jc.2015-2022 PMC4570160

[48] Castellano E, Attanasio R, Gianotti L, Cesario F, Tassone F, Borretta G (2016) Forearm DXA increases the rate of patients with asymptomatic primary hyperparathyroidism meeting surgical criteria. J Clin Endocrinol Metab 101(7):2728–2732. 10.1210/jc.2016-1513 27070376 10.1210/jc.2016-1513

